# Dietary Knowledge and Eating Habits among Patients with Type 2 Diabetes in Lebanon

**DOI:** 10.1155/2024/3623555

**Published:** 2024-02-06

**Authors:** Myriam Abboud, Cyrille Nacouzi, Zeina Chahine, Angelica Atallah, Mira Hleyhel

**Affiliations:** ^1^Department of Health, College of Natural and Health Sciences, Zayed University, Dubai 144534, UAE; ^2^Nutrition Department, Faculty of Public Health II, Lebanese University, Fanar, Lebanon; ^3^INSPECT-LB: Institut National de Santé Publique, Epidémiologie Clinique et Toxicologie, Beirut, Lebanon

## Abstract

Little is known about the dietary knowledge (DK) and eating habits (EHs) of patients with type 2 diabetes (T2D) in Lebanon. Therefore, the aim of this study was to assess the DK and EH of the population with T2D and determine their associated factors. A cross-sectional survey enrolling 351 patients with T2D was carried out, using the snowball sampling technique. The survey used the UK Diabetes and Diet Questionnaire and the Dietary Knowledge questionnaire to assess participants' EH including the frequency of consumption of certain foods and their knowledge of food groups and food choices. While a higher DK index indicated better knowledge, a higher EH index indicated less healthy EH. Independent sample *T*-test and Mann–Whitney test were used for dichotomous variables, and ANOVA and Kruskal–Wallis tests were used for polytomous variables. Correlation analysis tested the association between two continuous variables. Two multiple linear regression models were used to identify factors associated with DK and EH. Overall, 67% of participants had good or adequate DK, and around 25% and 75% of them had healthy and less healthy EH, respectively. Better knowledge was significantly related to occupation, BMI, presence of comorbidities, and HbA1c testing during the last 3 months. Higher family income, physical activity, family history of diabetes, receiving help in medication administration from family or friends, and higher DK level were factors associated with healthier EH. Nutrition education and awareness campaigns aimed at patients and their families are needed to empower patients with adequate DK and skills to facilitate the adoption of healthy EH.

## 1. Introduction

Diabetes is considered one of the global health emergencies of the 21^st^ century, affecting over 537 million adults worldwide in 2021, and its prevalence is estimated to rise to 783 million by 2045 [1]. In 2019, the world's highest diabetes prevalence was reported in the Middle East and North Africa (MENA) region at 12.2% [2]. In Lebanon, a country in the Eastern Mediterranean basin, the prevalence of type 2 diabetes (T2D) is estimated to be 7.95% among adults [3] and 15% among adults in Beirut alone [4], with an incidence of 17.2 per 1000 person-years which stands on the high side for the MENA region [5]. Poor HbA1c and blood pressure control, unhealthy lipid profiles, and physical inactivity are common among patients with T2D in Lebanon [6], and diabetes complications affect 22% of them [3].

With this alarming increase, and in order to decrease complications and diabetes-related mortality, this health problem requires a comprehensive management plan where patients should be educated to make informed decisions about diet, exercise, weight, and medications, as recommended by the American Diabetes Association (ADA) [7]. In fact, patient education has been proven to improve knowledge, dietary behaviors, and health outcomes in patients with T2D, including HbA1c and mortality reductions, lower weight and body fat, and improved quality of life [8]. Also, knowledge of diabetes was found to be associated with compliance with treatment and a decrease in complications [9]. In Jordan [10] and Nigeria [11], studies reported poor dietary knowledge (DK) levels in the population with T2D, thus hindering diabetes outcomes. In Lebanon, studies showed that patients' knowledge and practice scores related to diabetes' self-management were unsatisfactory [12]. However, a diabetes education program helped improve the glycemic levels, dietary habits, body anthropometrics, and lipid profile of Lebanese patients with T2D [13].

In addition to genetics, the economic development and urbanization, the nutrition transition, and the subsequent divergence from the Mediterranean diet toward a more westernized diet are all important factors underlying the increase in diabetes prevalence in the MENA region generally and in Lebanon specifically [14]. Individuals' eating habits (EHs) are now characterized by increased consumption of refined carbohydrates, added sugar, fats, and processed and animal source foods along with reduced fruit and vegetable intake and physical inactivity [15, 16].

Given the increasing prevalence of T2D, the poor EH, and the deficient knowledge in terms of diabetes management among patients with diabetes in Lebanon, and since studies investigating DK and current dietary habits in the population in Lebanon are still lacking, the main objectives of our study were to assess the DK and the EH of patients with T2D in Lebanon and to identify the factors associated with both variables.

## 2. Materials and Methods

### 2.1. Study Design and Population

This is a cross-sectional study conducted between February and June 2021, assessing the DK and EH of patients with T2D in Lebanon.

Eligible participants were patients with self-reported T2D (based on the answer to the question Are you diagnosed with T2D?), aged between 18 and 85 years, and currently living in Lebanon. Patients with type 1 diabetes (T1D) were excluded.

### 2.2. Sampling

Snowball sampling was used to identify potential study participants. The online questionnaire was sent to relatives, friends, colleagues, dietitians, and diabetes organizations in Lebanon (Chronic Care Center, DiaLeb, and Lebanese Diabetes Society) who were asked to spread the questionnaire to potential participants.

### 2.3. Survey Instrument

The survey instrument was a self-administered online questionnaire of four sections. The first section collected data about sociodemographic characteristics, lifestyle behaviors, and the personal and family medical histories of the participants. The second section gathered information related to T2D, such as its duration, blood glucose and glycosylated hemoglobin type A1c (HbA1c) testing, and the presence of complications. The UK Diabetes and Diet Questionnaire [17] was used in the third section to assess the EH such as the consumption frequency of different types of common food groups over the last month. Some of the questions were slightly adapted to the national and cultural Lebanese context by adding some examples of Lebanese foods. The last section used the Dietary Knowledge questionnaire from Sami et al. [18] to assess patients' DK about food groups, types, and choices.

The questionnaire was developed in English, and a translation to Arabic was performed by a certified translator to ensure the participation of subjects from different educational backgrounds. Back translation was performed to ensure linguistic and cross-cultural validity of the study. Both the English and Arabic versions were evaluated for consistency in meaning by bilingual experts. The reliability of individual questions within the DK and EH questionnaires was examined, and the Cronbach value was 0.6 for the correlation of questions within both questionnaires.

### 2.4. Ethical Approval and Consent to Participate

This study was approved by the Research Ethics Committee (REC) at INSPECT-LB, Lebanon (2022REC-004-INSPECT-02-15). Each participant received a full disclosure of the nature and purpose of the study, was reassured of the confidentiality of the data, and was given the opportunity to ask questions. Online written informed consent was obtained from all subjects.

### 2.5. Statistical Analysis

DK and EH indices were calculated. For the DK index, each question had one correct answer and each correct answer was given one point, whereas wrong or “I do not know” answers were given zero points. For each participant, correct answers were summed to obtain an index ranging between 0 and 21. The higher the index, the greater the knowledge level. The DK index was also converted into percentage and classified into three levels of knowledge: poor DK (<50%), good DK (between 50% and 75%), or adequate DK (>75%) [18].

For the EH index, single select multiple choice questions were included. Each question had a set of six choices (A, B, C, D, E, and F): A and B answers reflected a healthy dietary choice, C and D reflected a less healthy dietary choice, and E and F reflected an unhealthy dietary choice. The responses were coded as 5 points for F, 4 for E, 3 for D, 2 for C, 1 for B, and 0 for A [19]. The EH index was calculated by summing all the answers. It ranged between 0 and 115 and was classified into three levels according to Bloom's cutoff points: healthy EH (0 to 38), less healthy EH (39 to 77), and unhealthy EH (78 to 115). The lower the index is, the healthier the EHs are.

To describe participants' characteristics, frequencies and percentages were used for categorical variables, and means and standard deviations were used for continuous variables. Two separate multiple linear regression models were used to identify factors associated with DK and EH. Variables that showed a *p* value <0.2 in the bivariate analysis were included in the regression models [20, 21]. The histogram and the Q-Q plots of both the dependent variables (DK and EH indices) and the residuals of the linear regressions were checked to verify the normality of the distribution. *p* < 0.05 was considered statistically significant. Statistical analyses were conducted using the Statistical Package for Social Sciences (SPSS) software, Version 25.

## 3. Results

### 3.1. Sociodemographic and Lifestyle Characteristics

The study included 351 participants with a mean age of 59.8 years. Most of the participants were Lebanese, recruited from all the Lebanese territories, particularly Mount Lebanon. More than half of the sample was married (64.7%), and about one-third achieved a university degree level. Approximately 18% of the participants in the survey had a BMI within the normal range.  Table 1 provides a summary of the sociodemographic and lifestyle characteristics of the participants.

### 3.2. Medical and Diabetes-Related Characteristics

Most participants reported having a family history of diabetes (84.6%), and less than half (39.3%) reported having at least one diabetes complication. A large proportion of participants did not monitor their blood glucose levels (45.0%) or test their HbA1c levels during the last three months (34.5%). Regarding the dietary management of T2D, less than 25% of the participants followed a diabetic-friendly diet.  Table 2 presents a description of participants' medical and diabetes-related characteristics, including monitoring, pharmacological, and dietary management of diabetes.

### 3.3. Dietary Knowledge

More than half of the sample acknowledged that the diabetic diet is healthy for most people (58.4%); however, only few were able to define a well-balanced diet (41%). Although a large proportion was able to recognize the effect of unsweetened fruit juice on blood glucose (66.1%) and the relationship between excessive sugar consumption and diabetes (88.6%), not many participants knew that hard candies can be used to treat low blood glucose levels (27.6%) and that HbA1c is strongly related to the quality of the diet (38.5%). Moreover, a large percentage (58.4%) wrongly believed that artificial sweeteners have the highest amount of sugar (58.4%) and that foods labeled “sugar-free” can be eaten freely (39.9%).

Participants had good knowledge of carbohydrates' food sources, including breads, cereals, rice, pasta (94%), and baked potatoes (64.7%), as well as of the high fiber content of whole grain foods (88.6%) and the high glycemic index of dextrose (60.7%). Respondents also reported correct answers regarding chicken and meat being among foods with the highest amounts of proteins (57.8%), and fish being a complete source of protein (68.4%). Overall, the average DK index was 12.03 over 21, reflecting a good average level of knowledge, with 33% of the participants having poor DK, 52% having good DK, and only 15% of them having adequate DK (Table 3).

The regression coefficients and 95% confidence intervals of the linear regression analysis, as presented in Table 4, show the relationships between participants' characteristics and the DK index. DK was greater among retired individuals (*β* = 1.379 (95% CI: 0.144 to 2.613); *p*=0.029) and lower among those living outside Beirut (*β* = −1.340 (95% CI: −2.308 to −0.373); *p*=0.007). Being overweight (*β* = 1.005 (95% CI: 0.191 to 1.820); *p*=0.019) or obese (*β* = 1.632 (95% CI: 0.765 to 2.499); *p* < 0.001), drinking alcohol (*β* = 0.931 (95% CI: 0.151 to 1.711); *p*=0.020), having at least one comorbidity (*β* = 0.075 (95% CI: 0.024 to 0.126); *p*=0.004), and having had HbA1c tested during the last 3 months (*β* = 0.829 (95% CI: 0.243 to 1.415); *p*=0.006) were associated with higher DK.

### 3.4. Eating Habits

More than 30% of the respondents reported typically consuming at least three regular meals a day (33.6%) and having breakfast within two hours of waking up, 5 to 6 times per week (30.5%). While 57.5% of them ate one to two portions of bread per day and 49% ate a serving of pasta or rice per week, the majority had never consumed high-fiber bread (53.8%) and high-fiber pasta or rice (70.4%). Savory foods (35%), sweet pastries (43.3%), savory pastries (41.3%), desserts (41.9%), and high fat/sugar snacks (37.9%) were consumed once per week or less often, whereas fast foods (48.1%), sweets (43%), and sugary drinks (48.1%) were never consumed by most of the participants. In addition, almost 40% of patients never consumed fatty fish (37.2%). Also, nearly half of them consumed vegetables (49.6%) and fruits (44.4%) 5 to 6 times per week.

Overall, most of the participants (75%) had less healthy EH, compared to only 25% adopting a healthy diet, with the average of the EH index being 43.81 over 115 (Table 3).

A linear regression analysis, as presented in Table 5, showed that alcohol consumption (*β* = 6.521 (95% CI: 3.935 to 9.108); *p* < 0.001) and checking the nutritional composition of foods (*β* = 3.088 (95% CI: 1.018 to 5.157); *p*=0.004) were significantly associated with less healthy EH. However, EHs were healthier among retired individuals (*β* = −5.122 (95% CI: −9.008 to −1.236); *p*=0.010) and among those who reported higher household income (*β* = −4.132 (95% CI: −7.448 to −0.816); *p*=0.015). Moreover, those who reported having a family history of diabetes (*β* = −3.804 (95% CI: −6.049 to −1.558); *p*=0.001), being physically active (*β* = −3.280 (95% CI: −6.030 to −0.529); *p*=0.020), and having higher DK (*β* = −0.690 (95% CI: −1.051 to −0.330); *p* < 0.001) were more likely to have healthy EH.

## 4. Discussion

To our knowledge, this study is the first to explore the DK and EH of patients with T2D in Lebanon. Around 67% (*n* = 235) of the participants had good or adequate DK. Better knowledge was significantly related to occupation (*p*=0.029), BMI (*p* < 0.001), presence of comorbidities (*p*=0.004), and HbA1c testing (0.006) (Table 4). Around 25% and 75% of the participants reported healthy and less healthy EH, respectively. Higher family income (*p*=0.015), physical activity (*p*=0.020), family history of diabetes (*p*=0.001), and higher DK level (*p* < 0.001) were factors significantly associated with healthier EH among patients with T2D in this study (Table 5).

In terms of DK, our findings were similar to those of studies conducted in Sudan [22] and Iran [23] where more than half of the participants with T2D had good DK levels. A different pattern of results was observed in the Kingdom of Saudi Arabia (KSA) [18], Jordan [10], and Nigeria [11] where subjects demonstrated poor DK. The difference in knowledge levels among these populations with T2D may be attributable to the different knowledge assessment tools, study designs, and sociocultural differences between these populations. One of the interpretations of high knowledge levels among our participants is that a large proportion of those with good to adequate DK (42.5%) were university degree holders.

Among our participants, university degree holders account for 42.5% of those with good knowledge, which may contribute to the high knowledge levels.

The average BMI among T2D patients in this study was 28.7 kg/m^2^, which was concordant with previous reports where most T2D patients in Lebanon had a BMI between 25 and 29 or above [5, 12]. The association between higher BMI and better DK found here was in accordance with studies in the KSA showing that overweight and obese individuals had better diabetes knowledge than their normal-weight peers [24, 25]. Our study also showed that the presence of comorbidities and regular HbA1c testing were associated with better DK. It is expected that patients of higher weights, those with concomitant diseases, or those who regularly test their HbA1c levels can become increasingly concerned about their health, which incites them to deepen their knowledge and understanding of their medical conditions, enabling them to develop healthy habits. Moreover, the association between being retired and higher DK knowledge levels may be attributed to time availability, increased self-care, and desire for wellness, as well as the access to resources and social engagement that accompany retirement [26], leading retirees to deepen their knowledge about proper nutrition and healthier EH.

Concerning EH, our study showed similar results to prior research on EH conducted in Ethiopia [27] and the UAE [28], which found that T2D patients' diet was of poor quality. This result is likely to be related to the significant changes in EH toward western eating patterns. In fact, a recent review of studies showed a trend of decreasing adherence to the Mediterranean diet in Lebanon over time [29]. Our findings also showed that of those who have less healthy EH, 19% were in debt, owing money that needs to be repaid, and around 60% were meeting routine expenses only. Therefore, another possible explanation of poor dietary quality, especially among low-income earners, is severe food insecurity, with more than 75% of the Lebanese currently living below the poverty line, and around 34% being food insecure [30]. In regard to the factors associated with EH, our results build on existing evidence [31, 32] showing that lifestyle behaviors, including alcohol consumption and physical inactivity, are associated with less healthy EH. On the other hand, having a family history of diabetes was more likely to lead to improved EH, which accords with other studies' findings showing that patients with a family history of diabetes were more likely to engage in healthy EH [33, 34]. Another interesting result is the association between checking nutritional labels and less healthy EH, which demonstrates, similarly to another study [35], that the use of food labels does not necessarily improve dietary quality or EH. Misusing and misunderstanding food labels were shown to be barriers to information and to making healthy eating decisions [36]. Even though the DK level was satisfactory for most participants and DK was a predictor of EH, nearly 75% of our patients had less healthy EH. This result suggests that barriers to knowledge application and patient adherence to dietary guidelines are present and that better strategies are needed to help patients achieve their dietary and diabetes goals.

According to our results, family members play a considerable role in diabetes care, medication administration, and dietary intake. Therefore, interactive and individualized sessions involving physicians and dietitians, with the family present, can teach and motivate patients and their families and provide them with tips for real-world application of diet recommendations, thereby improving diabetes outcomes. Moreover, awareness campaigns aimed at patients and their families can help promote awareness in a creative and informative way and improve knowledge of the diabetic diet.

### 4.1. Limitations

Several limitations of the study should be noted, including the use of snowball sampling, which could have resulted in reducing the representativeness of our study. However, this was partly compensated by including patients with T2D from the different Lebanese regions. While a Cronbach alpha >0.6 is desirable in terms of internal consistency, the study provided transparency regarding the specific items included in the questionnaires. The selected parameters were clinically relevant and captured meaningful aspects of DK and EH.

In relation to the validity of the research instrument, the DK questionnaire developed by Sami et al. was validated for assessing patients' knowledge about carbohydrates, lipids, proteins, food types, and food choices among patients with T2D in KSA and showed good internal consistency reliability [18]. Similarly, the EH questionnaire used in this study was also a valid and reliable tool for assessing the EH of patients with T2D in the UK [19]. While we recognize the distinctiveness of the Lebanese dietary patterns, it is noteworthy that general questions collecting information about common food types and groups may contribute to the questionnaires' applicability in the Lebanese context. Future studies should prioritize the validation of the questionnaires specifically within the Lebanese population.

To our knowledge, our study was the first to provide some valuable insights into the DK levels and EH of patients with T2D in Lebanon. In this survey, and as it is frequently found, females were more willing to participate than men; however, this does not likely impact the results of the study since there were no significant associations between gender and DK or EH. Even though most participants were aged 50 years and older, we believe that a web-based survey is still feasible in this population, which is in agreement with what has been shown in a previous study [37], especially in Lebanon, where more than 89% of the total population has access to the Internet [38]. On the other hand, with participants having access to the Internet and receiving help from friends and family members, the data collected might not accurately reflect the level of respondents' DK. In addition, given the observational nature of our study, it is susceptible to information bias arising from underreporting of foods with a negative health image and overreporting of healthy EH and foods with a positive healthy image.

## 5. Conclusions

An appropriate diabetic diet for managing and controlling carbohydrate intake is considered one of the cornerstones of blood glucose control and overall health management in subjects with T2D. The results of the present study suggest that nutrition education reinforcement is needed, not only to empower patients with T2D with knowledge and skills to make the right food choices but also to facilitate the adoption of healthy EH.

Nutrition education sessions, proper educational tools, and awareness campaigns, led by a multidisciplinary team of healthcare professionals, can teach patients and their families how to manage the disease, reduce its symptoms, and prevent complications through proper dietary management. As awareness spreads, more individuals in the community with diabetes begin to seek answers and take action, which can have far-reaching benefits beyond patients with T2D.

## Figures and Tables

**Table 1:** Sociodemographic and lifestyle characteristics of patients with T2D included in the study (*n* = 351).

	Mean (*n*)	Standard deviation (%)
*Age (years)*	59.27	10.3

*Gender*		
Male	123	35
Female	228	65

*Nationality*		
Lebanese	315	89.7
Others	36	10.3

*Residence*		
Beirut	36	10.3
Outside Beirut^*∗*^	315	89.7

*Education level*		
High school	223	63.5
Graduate degree	108	30.8
Postgraduate degree and above	20	5.7

*Occupation*		
Nonworker	53	15.1
Housewife	108	33.6
Healthcare worker	8	2.3
Non-healthcare worker	149	42.5
Retired	23	6.6

*Family income*		
In debt	54	15.4
Just meet routine expenses	196	55.8
Meet routine expenses and emergencies	90	25.6
Able to save/invest money	11	3.1

*Body mass index (kg/m* ^*2*^)		
Underweight (<18.5)	1	0.3
Normal weight (18.5 to 24.9)	64	18.2
Overweight (25 to 29.9)	166	47.3
Obese (≥30)	120	34.2

*Physical activity*		
No	204	58.1
Less than 30 minutes per week	80	22.8
1 to 4 hours per week	61	17.4
More than 4 hours per week	6	1.7

*Smoking*		
Nonsmoker	206	58.7
Former smoker	95	27.1
Smoker	50	14.2

*Alcohol consumption*		
No	258	73.5
Occasionally (1 to 2 times per week)	69	19.7
More than 3 times per week	24	6.8

Summary statistics are expressed as mean and standard deviation for continuous variables and as frequency and percentage for categorical variables. ^*∗*^Regions outside Beirut include Mount Lebanon (*n* = 90, 25.6%), North Lebanon (*n* = 92, 26.3%), South Lebanon (*n* = 67, 19.1%), and Bekaa (*n* = 66, 18.9%).

**Table 2:** Medical and diabetes-related characteristics of patients with T2D included in the study (*n* = 351).

	Mean (*n*)	Standard deviation (%)
*Duration of diabetes (years)*	9.75	8.5
Missing (*n* = 30)		

*Family history of diabetes*		
No	54	15.4
Yes	269	84.6

*Presence of diabetes complications* ^*∗*^		
No	213	60.7
Yes	138	39.3

*Presence of other chronic diseases* ^*∗∗*^		
No	180	51.3
Yes	171	48.7

*Lipid lowering medications*		
No	257	73.2
Yes	94	26.8

*HbA1c testing during the last 3 months*		
Did not test	121	34.5
Tested but did not know the level	65	18.5
Tested and knew the level	165	47.0

*Daily blood glucose testing by fingerprick*		
No	158	45.0
Once	162	46.2
1 to 4 times	23	6.6
More than 4 times	8	2.3

*Checking nutritional composition of foods*		
No	253	72.1
Yes	98	27.9

*Responsible for diabetes care*		
Patient himself	81	23.1
Family member	137	39.0
Physician	133	37.9

*Help from family/friends in medication administration*		
No	132	37.6
Yes	219	62.4

*Help from family/friends in dietary intake*		
No	154	43.9
Yes	197	56.1

*Source of dietary knowledge*		
Physician	126	35.9
Dietitian	35	10.0
Diabetes organizations	58	16.5
Social media	188	53.6

Summary statistics are expressed as mean and standard deviation for continuous variables and as frequency and percentage for categorical variables. ^*∗*^Among diabetes complications reported, cardiovascular disease accounted for 27.9% (n = 98), kidney problems for 6.3% (n = 22), peripheral problems for 6.6% (n = 23), dental problems for 4.0% (n = 14), and nerve damage and pain for 1.7% (n = 6). Among chronic diseases reported, liver disease accounted for 1.1% (n = 4), cancer for 6.3% (n = 22), cardiovascular diseases for 21.9% (n = 77), dyslipidemia for 16.8% (n = 59), renal disease for 8.0% (n = 28), and hypertension for 39.6% (n = 139).

**Table 3:** Distribution of dietary knowledge and eating habit indices of patients with T2D included in the study (*n* = 351).

Index	Categories	*n*	%	Mean	SD	Median	Q1	Q3	Min	Max
DK index	<10.5: poor DK	116	33							
	10.5–15.75: good DK	184	52.4	12.0	2.9	12	10	14	4	21
	>15.75: adequate DK	51	14.5							

EH index	0–38: strong healthy EH	88	25.1							
	39–77: less healthy EH	263	74.9	43.8	9.1	44	38	49	18	72
	78–115: unhealthy EH	0	0.0							

EH, eating habit; DK, dietary knowledge; SD, standard deviation.

**Table 4:** Multiple linear regression for the factors associated with dietary knowledge index (*n* = 351).

	Dietary knowledge index				
	Unstandardized *β*^†^	Standardized *β*	Lower 95% CI of *β*	Upper 95% CI of *β*	*p* value
*Residence*					
Beirut	Ref	Ref	Ref	Ref	Ref
Outside Beirut	−1.3	−0.1	−2.3	−0.3	**0.007** ^*∗*^

*Occupation*					
Nonworker/housewife	Ref	Ref	Ref	Ref	Ref
Healthcare worker	0.9	0.9	−0.9	2.7	0.328
Non-healthcare worker	0.4	0.0	−0.2	1.1	0.183
Retired	1.3	0.1	0.1	2.6	**0.029** ^*∗*^

*BMI*					
Underweight/normal	Ref	Ref	Ref	Ref	Ref
Overweight	1.1	0.1	0.1	1.8	**0.016** ^*∗*^
Obese	1.6	0.2	0.7	2.4	**<0.001** ^*∗*^

*Alcohol consumption*					
No	Ref	Ref	Ref	Ref	Ref
Yes	0.9	0.1	0.1	1.7	**0.020** ^*∗*^

*Presence of other chronic diseases*					
No	Ref	Ref	Ref	Ref	Ref
Yes	0.1	0.1	0.1	0.1	**0.004** ^*∗*^

*HbA1c testing*					
No	Ref	Ref	Ref	Ref	Ref
Yes	0.8	0.1	0.2	1.4	**0.006** ^*∗*^

*Responsible for diabetes care*					
Physician	Ref	Ref	Ref	Ref	Ref
Patient himself or family/friend	2.3	0.3	1.4	3.3	**<0.001** ^*∗*^

^†^ *β*, regression coefficient. ^*∗*^*p* value <0.05: there is a linear relationship between independent variable and DK index adjusting for the effects of other variables. The analysis included age, nationality, residence (Beirut/outside Beirut), education level, occupation, family income, BMI, presence of other chronic diseases, alcohol consumption, smoking, duration of diabetes, daily blood glucose monitoring, HbA1c testing, lipid lowering medications use, responsible for diabetes care (patient himself or family member/physician), help in dietary intake, checking nutritional composition of foods, and social media and physician as sources of dietary information. *R*^2^ = 45.6%: 45.6% of the DK index is predicted by independent variables. The bold values in the table indicate p values that are less than 0.05, signifying statistical significance.

**Table 5:** Multiple linear regression for the factors associated with eating habit index (*n* = 351).

	Eating habit index				
	Unstandardized *β*^†^	Standardized *β*	Lower 95% CI of *β*	Upper 95% CI of *β*	*p* value
*Occupation*					
Nonworker/housewife	Ref	Ref	Ref	Ref	Ref
Healthcare worker	−1.9	−0.1	−4.1	0.3	0.092
Non-healthcare worker	−5.0	−0.01	−10.9	0.8	0.092
Retired	−5.1	−0.1	−9.1	−1.2	**0.010** ^*∗*^

*Family income*					
In debt	Ref	Ref	Ref	Ref	Ref
Just meet routine expenses	0.1	0.1	−2.4	2.7	0.916
Meet routine expenses and emergencies	−4.1	−0.2	−7.4	−0.8	**0.015** ^*∗*^
Able to save/invest money	3.5	0.1	−2.2	9.3	0.228

*Physical activity*					
No	Ref	Ref	Ref	Ref	Ref
Yes	−3.2	−0.1	−6.0	−0.5	**0.020** ^*∗*^

*Alcohol consumption*					
No	Ref	Ref	Ref	Ref	Ref
Yes	6.5	0.3	3.9	9.1	**<0.001** ^*∗*^

*Family history of diabetes*					
No	Ref	Ref	Ref	Ref	Ref
Yes	−3.8	−0.1	−6.1	−1.5	**0.001** ^*∗*^

*Help in medication administration*					
No	Ref	Ref	Ref	Ref	Ref
Yes	−1.9	−0.1	−3.8	−0.1	**0.049** ^*∗*^

*Responsible for diabetes care*					
Physician	Ref	Ref	Ref	Ref	Ref
Patient himself or family/friend	4.9	0.2	2.1	7.9	**0.001** ^*∗*^

*Checking nutritional composition of foods*					
No	Ref	Ref	Ref	Ref	Ref
Yes	3.1	0.1	1.1	5.1	**0.004** ^*∗*^

*DK score*	−0.6	−0.2	−1.1	−0.3	**<0.001** ^*∗*^

^†^ *β*, regression coefficient. ^*∗*^*p* value <0.05: there is a linear relationship between independent variable and DK index adjusting for the effects of other variables. The analysis included age, gender, nationality, residence (Beirut/outside Beirut), education level, occupation, family income, BMI, presence of other chronic diseases, presence of diabetes complications, family history of diabetes, physical activity, alcohol consumption, smoking, HbA1c testing, responsible for diabetes care (patient himself or family member/physician), help in medication administration, checking nutritional composition of foods, social media, physician, and dietitian as sources of dietary information, and DK score. Being physically active refers to engaging in more than one hour of physical activity per week. *R*^2^ = 41.7%: 41.7% of the EH index is predicted by independent variables. The bold values in the table indicate p values that are less than 0.05, signifying statistical significance.

## Data Availability

The datasets analysed during the current study are available from the corresponding author upon reasonable request.
